# Supplementary material for: Adsorption of Phenol and Chlorophenols by HDTMA Modified Halloysite Nanotubes
Source: Materials (Basel). 2020 Jul 24;13(15):3309. doi: 10.3390/ma13153309 (PMC7436260; doi:10.3390/ma13153309)
Supplement: Supplementary file 1 [file materials-13-03309-s001.pdf]

Supplementary Materials

Adsorption of Phenol and Chlorophenols by HDTMA Modified Halloysite Nanotubes

Piotr M. Słomkiewicz [1,*], Beata Szczepanik [1] and Marianna Czaplicka [2]

[1] Institute of Chemistry, Jan Kochanowski University, 7 Uniwersytecka, 25-406 Kielce, Poland; Beata.Szczepanik@ujk.edu.pl

[2] Institute of Environmental Engineering Polish Academy of Sciences, 34 M. Skłodowskiej-Curie St., 41-819 Zabrze, Poland; marianna.czaplicka@ipis.zabrze.pl

* Correspondence: piotres@ujk.edu.pl; Tel./Fax: +48-413497005

Received: 5 June 2020; Accepted: 21 July 2020; Published: date

Table S1. Adsorption isotherms data for adsorbates at 298 K on adsorbent HDTMA/HAL.

PH		2CPH		3CPH	
c_i	a_i	c_i	a_i	c_i	a_i
0	0	0	0	0	0
0.00342	6.82565×10^{-4}	0.00347	3.22876×10^{-4}	1.95361×10^{-4}	3.8429×10^{-5}
0.00679	0.00136	0.00692	5.61273×10^{-4}	3.89271×10^{-4}	6.31287×10^{-5}
0.01009	0.00201	0.01036	7.74393×10^{-4}	5.85285×10^{-4}	8.46721×10^{-5}
0.01346	0.00269	0.01388	9.78679×10^{-4}	7.78398×10^{-4}	1.03966×10^{-4}
0.01684	0.00336	0.01736	0.00117	9.72816×10^{-4}	1.22068×10^{-4}
0.02023	0.00403	0.02074	0.00135	0.00117	1.3922×10^{-4}
0.02363	0.0047	0.02423	0.00153	0.00136	1.55495×10^{-4}
0.02701	0.00537	0.02769	0.0017	0.00156	1.71194×10^{-4}
0.03025	0.00601	0.03111	0.00187	0.00175	1.86356×10^{-4}
0.03362	0.00668	0.03462	0.00203	0.00195	2.0102×10^{-4}
0.03702	0.00735	0.03807	0.00219	0.00214	2.15325×10^{-4}
0.04034	0.008	0.04161	0.00235	0.00233	2.29198×10^{-4}
0.04372	0.00867	0.04508	0.00251	0.00253	2.42825×10^{-4}
0.04711	0.00933	0.04888	0.00267	0.00273	2.5625×10^{-4}
0.05049	0.01	0.05214	0.00282	0.00293	2.70118×10^{-4}
0.05383	0.01065	0.05576	0.00297	0.00311	2.81928×10^{-4}
0.05716	0.0113	0.05884	0.0031	0.00331	2.94586×10^{-4}
0.06055	0.01196	0.06274	0.00326	0.0035	3.06898×10^{-4}
0.06406	0.01265	0.06563	0.00338	0.0037	3.19057×10^{-4}
0.0673	0.01328	0.06904	0.00352	0.00389	3.31055×10^{-4}

4–CPH		2.4–DCPH		2.4.6–TCPH	
c_i	a_i	c_i	a_i	c_i	a_i
0	0	0	0	0	0
4.62863×10^{-4}	3.61764×10^{-4}	0.00287	3.72787×10^{-4}	0.00195	3.92384×10^{-4}
9.26559×10^{-4}	5.79824×10^{-4}	0.00572	6.66485×10^{-4}	0.00387	6.34532×10^{-4}
0.00139	7.62321×10^{-4}	0.0086	9.3825×10^{-4}	0.00575	8.37224×10^{-4}
0.00185	9.26288×10^{-4}	0.01144	0.00119	0.00768	0.00102
0.00231	0.00108	0.0143	0.00144	0.00961	0.0012
0.00277	0.00122	0.01717	0.00167	0.01153	0.00136
0.00323	0.00135	0.0201	0.00191	0.0135	0.00152
0.00369	0.00148	0.02299	0.00214	0.01533	0.00166
0.00415	0.00161	0.02584	0.00236	0.01727	0.00181
0.00462	0.00173	0.0286	0.00257	0.01919	0.00194
0.00508	0.00184	0.03157	0.00279	0.02113	0.00208
0.00554	0.00195	0.0343	0.00299	0.02307	0.00221
0.006	0.00206	0.03739	0.00322	0.02491	0.00233
0.00646	0.00217	0.04008	0.00341	0.02689	0.00246
0.00692	0.00227	0.04305	0.00362	0.0288	0.00258
0.00738	0.00237	0.04594	0.00382	0.03072	0.0027
0.00784	0.00247	0.04862	0.00401	0.03259	0.00281
0.00831	0.00257	0.05156	0.00421	0.03452	0.00293
0.00877	0.00267	0.05452	0.00441	0.03642	0.00304
0.00923	0.00276	0.05717	0.00459	0.03832	0.00315
